# 4-Methyl-3-(2-phenoxy­acet­yl)-5-phenyl-1,3,4-oxadiazinan-2-one

**DOI:** 10.1107/S1600536809020285

**Published:** 2009-06-06

**Authors:** Julio Zukerman-Schpector, Lucas Sousa Madureira, Alessandro Rodrigues, Elisângela Vinhato, Paulo R. Olivato

**Affiliations:** a Universidade Federal de São Carlos, Laboratório de Cristalografia, Estereodinâmica e, Modelagem Molecular, Departamento de Química, 13565-905 São Carlos, SP, Brazil; b Universidade de São Paulo, Conformational Analysis and Electronic Interactions, Laboratory, Instituto de Química, São Paulo, SP, Brazil

## Abstract

The 1,3,4-oxadiazinane ring in the title compound, C_18_H_18_N_2_O_4_, is in a twisted boat conformation. The two carbonyl groups are orientated towards the same side of the mol­ecule. The dihedral angle between the planes of the benzene rings is 76.6 (3)°. Mol­ecules are sustained in the three-dimensional structure by a combination of C—H⋯O, C—H⋯π and π–π [shortest centroid–centroid distance = 3.672 (6) Å] inter­actions.

## Related literature

For synthetic and structural studies of substituted heterocyclic rings, see: Rodrigues *et al.* (2006 ▶). For puckering parameters, see: Cremer & Pople (1975 ▶); Iulek & Zukerman-Schpector (1997 ▶). For the synthesis, see: Rodrigues *et al.* (2005 ▶).
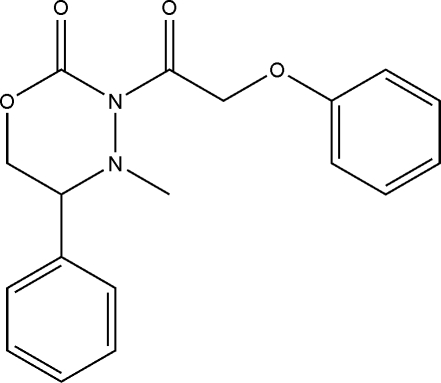


## Experimental

### 

#### Crystal data

C_18_H_18_N_2_O_4_
*M* *_r_* = 326.34
Monoclinic, 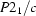

*a* = 9.6024 (9) Å
*b* = 9.4203 (10) Å
*c* = 19.275 (3) Å
β = 114.206 (9)°
*V* = 1590.3 (4) Å^3^
*Z* = 4
Mo *K*α radiation
μ = 0.10 mm^−1^
*T* = 290 K
0.15 × 0.10 × 0.08 mm

#### Data collection

Enraf–Nonius CAD-4 diffractometer
Absorption correction: none
2971 measured reflections
2793 independent reflections
1355 reflections with *I* > 2σ(*I*)
*R*_int_ = 0.030
3 standard reflections frequency: 60 min intensity decay: <1%

#### Refinement

*R*[*F* ^2^ > 2σ(*F* ^2^)] = 0.064
*wR*(*F* ^2^) = 0.246
*S* = 1.12
2793 reflections
218 parameters
H-atom parameters constrained
Δρ_max_ = 0.19 e Å^−3^
Δρ_min_ = −0.23 e Å^−3^

### 

Data collection: *CAD-4 EXPRESS* (Enraf–Nonius, 1994 ▶); cell refinement: *CAD-4 EXPRESS*; data reduction: *XCAD4* (Harms & Wocadlo, 1995 ▶); program(s) used to solve structure: *SIR97* (Altomare *et al.*, 1999 ▶); program(s) used to refine structure: *SHELXL97* (Sheldrick, 2008 ▶); molecular graphics: *ORTEP-3 for Windows* (Farrugia, 1997 ▶); software used to prepare material for publication: *WinGX* (Farrugia, 1999 ▶), *PARST* (Nardelli, 1995 ▶) and *MarvinSketch* (ChemAxon, 2008 ▶).

## Supplementary Material

Crystal structure: contains datablocks global, I. DOI: 10.1107/S1600536809020285/tk2466sup1.cif

Structure factors: contains datablocks I. DOI: 10.1107/S1600536809020285/tk2466Isup2.hkl

Additional supplementary materials:  crystallographic information; 3D view; checkCIF report

## Figures and Tables

**Table 1:** Hydrogen-bond geometry (Å, °)

*D*—H⋯*A*	*D*—H	H⋯*A*	*D*⋯*A*	*D*—H⋯*A*
C7—H7⋯O4^i^	0.93	2.53	3.394 (7)	154
C9—H9⋯O3^ii^	0.93	2.61	3.383 (8)	141
C13—H13⋯*Cg*2^iii^	0.93	2.86	3.715 (6)	153
C18—H18*B*⋯*Cg*3^i^	0.96	2.74	3.672 (6)	165

Symmetry codes: (i) 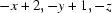; (ii) 
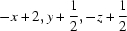
; (iii) 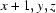. *Cg*2 is the centroid of the C6–C11 ring and *Cg*3 is the centroid of the C12–C17 ring.

**Fig. 1.**
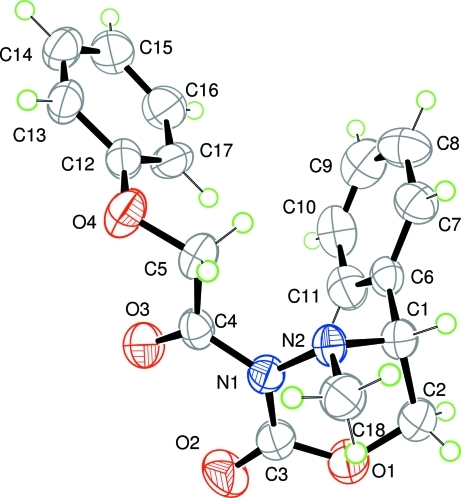
The molecular structure of (I) showing atom labelling scheme and displacement ellipsoids at the 50% probability level (arbitrary spheres for the H atoms).

## supplementary crystallographic information

### Comment

In continuation of synthetic and structural studies of substituted heterocyclic rings (Rodrigues *et al.*, 2006), the title compound (I) was prepared. The 1,3,4-oxadiazinane ring in (I), Fig. 1, is in a distorted twist boat conformation with the distortion being towards a boat conformation. The ring-puckering parameters (Cremer & Pople, 1975; Iulek & Zukerman-Schpector, 1997) were calculated as q_2_ = 0.119 (6) Å, q_3_ = -0.496 (6) Å, Q = 0.510 (6) Å, and φ_2_ = -108 (3)°. The ring- and side-chain-bound carbonyl groups lie to the same side of the molecule. The dihedral angle between the phenyl rings is of 76.6 (3)°. Molecules are sustained in the 3-D structure by a combination of C-H···O and π–π interactions, Table 1.

### Experimental

The starting (*R*)-4-methyl-5-phenyl-1,3,4-oxadiazinan-2-one was synthesized by using a previously reported procedure (Rodrigues *et al.* 2005). The phenoxyacetyl-1,3,4-oxadiazinan-2-one derivative was prepared by an acylation reaction of 1,3,4-oxadiazinan-2-one (Rodrigues *et al.* 2005). To a mixture of 1,3,4-oxadiazinan-2-one (500 mg, 2.60 mmol), 4-dimethylaminopyridine (16 mg, 0.13 mmol) and 2-phenoxyacetic acid (435 mg, 2.86 mmol) in CH_2_Cl_2_ (4 ml) at 273 K, under a nitrogen atmosphere, N,N-Dicyclohexylcarbodiimide was added in one portion (590 mg, 2.86 mmol). The temperature of the resulting suspension was allowed to reach room temperature. Stirring was continued until no starting material was left, as confirmed by TLC (20 h). The dicyclohexylurea formed was filtered and the precipitate washed with CH_2_Cl_2_ (20 ml). The filtrate was washed with a saturated aqueous solution of NaHCO_3_ (15 ml) and dried over Na_2_SO_4_. Filtration and evaporation yielded the crude solid, which was purified by flash chromatography on silica gel (hexane-EtOAc, 6:4). Colourless crystals of (I) were obtained by vapour diffusion from hexane/chloroform at 298 K.

### Refinement

The H atoms were positioned with idealized geometry using a riding model with C—H = 0.93–0.98 Å, and with *U*_iso_ set to 1.2 times (1.5 for methyl) *U*_eq_(parent atom).

### Crystal data

**Table:**

C_18_H_18_N_2_O_4_	*F*(000) = 688
*M**_r_* = 326.34	*D*_x_ = 1.363 Mg m^−^^3^
Monoclinic, *P*2_1_/*c*	Melting point = 385–387 K
Hall symbol: -P 2ybc	Mo *K*α radiation, λ = 0.71073 Å
*a* = 9.6024 (9) Å	Cell parameters from 24 reflections
*b* = 9.4203 (10) Å	θ = 10.5–15.1°
*c* = 19.275 (3) Å	µ = 0.10 mm^−^^1^
β = 114.206 (9)°	*T* = 290 K
*V* = 1590.3 (4) Å^3^	Irregular, colourless
*Z* = 4	0.15 × 0.10 × 0.08 mm

### Data collection

**Table:**

Enraf–Nonius CAD-4 diffractometer	*R*_int_ = 0.030
Radiation source: fine-focus sealed tube	θ_max_ = 25.0°, θ_min_ = 2.3°
graphite	*h* = −11→0
ω–2θ scans	*k* = 0→11
2971 measured reflections	*l* = −20→20
2793 independent reflections	3 standard reflections every 60 min
1355 reflections with *I* > 2σ(*I*)	intensity decay: <1%

### Refinement

**Table:**

Refinement on *F*^2^	Primary atom site location: structure-invariant direct methods
Least-squares matrix: full	Secondary atom site location: difference Fourier map
*R*[*F*^2^ > 2σ(*F*^2^)] = 0.064	Hydrogen site location: inferred from neighbouring sites
*wR*(*F*^2^) = 0.246	H-atom parameters constrained
*S* = 1.12	*w* = 1/[σ^2^(*F*_o_^2^) + (0.0852*P*)^2^ + 2.8996*P*] where *P* = (*F*_o_^2^ + 2*F*_c_^2^)/3
2793 reflections	(Δ/σ)_max_ < 0.001
218 parameters	Δρ_max_ = 0.19 e Å^−^^3^
0 restraints	Δρ_min_ = −0.23 e Å^−^^3^

### Special details

**Table:**

Geometry. All s.u.'s (except the s.u. in the dihedral angle between two l.s. planes) are estimated using the full covariance matrix. The cell s.u.'s are taken into account individually in the estimation of s.u.'s in distances, angles and torsion angles; correlations between s.u.'s in cell parameters are only used when they are defined by crystal symmetry. An approximate (isotropic) treatment of cell s.u.'s is used for estimating s.u.'s involving l.s. planes.
Refinement. Refinement of *F*^2^ against ALL reflections. The weighted *R*-factor *wR* and goodness of fit *S* are based on *F*^2^, conventional *R*-factors *R* are based on *F*, with *F* set to zero for negative *F*^2^. The threshold expression of *F*^2^ > σ(*F*^2^) is used only for calculating *R*-factors(gt) *etc*. and is not relevant to the choice of reflections for refinement. *R*-factors based on *F*^2^ are statistically about twice as large as those based on *F*, and *R*- factors based on ALL data will be even larger.

### Fractional atomic coordinates and isotropic or equivalent isotropic displacement parameters (Å^2^)

**Table:**

	*x*	*y*	*z*	*U*_iso_*/*U*_eq_	
C1	0.6770 (6)	0.3140 (6)	−0.0050 (3)	0.0504 (14)	
H1	0.6177	0.3840	−0.0433	0.060*	
C2	0.5715 (6)	0.1861 (6)	−0.0126 (4)	0.0676 (18)	
H2A	0.5123	0.2040	0.0169	0.081*	
H2B	0.5008	0.1758	−0.0655	0.081*	
C3	0.8059 (6)	0.0406 (6)	0.0376 (3)	0.0530 (14)	
C4	1.0422 (5)	0.1778 (6)	0.0667 (3)	0.0411 (12)	
C5	1.0994 (5)	0.3123 (6)	0.0478 (3)	0.0492 (13)	
H5A	1.0756	0.3145	−0.0062	0.059*	
H5B	1.0487	0.3921	0.0593	0.059*	
C6	0.7359 (5)	0.3870 (6)	0.0714 (3)	0.0419 (12)	
C7	0.7468 (6)	0.5357 (6)	0.0737 (3)	0.0550 (14)	
H7	0.7155	0.5862	0.0284	0.066*	
C8	0.8025 (8)	0.6080 (7)	0.1415 (4)	0.0690 (18)	
H8	0.8055	0.7067	0.1416	0.083*	
C9	0.8541 (7)	0.5350 (9)	0.2091 (4)	0.0735 (19)	
H9	0.8956	0.5837	0.2551	0.088*	
C10	0.8439 (7)	0.3903 (8)	0.2083 (3)	0.0643 (17)	
H10	0.8769	0.3407	0.2539	0.077*	
C11	0.7850 (6)	0.3167 (7)	0.1400 (3)	0.0596 (16)	
H11	0.7784	0.2182	0.1405	0.071*	
C12	1.3081 (6)	0.3918 (6)	0.1593 (3)	0.0452 (12)	
C13	1.4569 (5)	0.4434 (6)	0.1878 (3)	0.0498 (14)	
H13	1.5174	0.4293	0.1612	0.060*	
C18	0.7580 (7)	0.2267 (6)	−0.1031 (3)	0.0593 (16)	
H18A	0.8470	0.2022	−0.1114	0.089*	
H18B	0.7058	0.3041	−0.1358	0.089*	
H18C	0.6912	0.1461	−0.1141	0.089*	
C14	1.5132 (6)	0.5148 (6)	0.2553 (3)	0.0573 (15)	
H14	1.6120	0.5508	0.2739	0.069*	
C15	1.4279 (7)	0.5343 (7)	0.2958 (3)	0.0640 (17)	
H15	1.4668	0.5849	0.3412	0.077*	
C16	1.2833 (7)	0.4782 (7)	0.2689 (3)	0.0650 (16)	
H16	1.2256	0.4878	0.2973	0.078*	
C17	1.2231 (6)	0.4082 (6)	0.2007 (3)	0.0544 (14)	
H17	1.1244	0.3719	0.1826	0.065*	
N1	0.8837 (4)	0.1580 (4)	0.0262 (2)	0.0420 (10)	
N2	0.8042 (4)	0.2689 (4)	−0.0233 (2)	0.0383 (10)	
O1	0.6553 (4)	0.0565 (4)	0.0129 (3)	0.0744 (13)	
O2	0.8648 (5)	−0.0680 (4)	0.0671 (3)	0.0712 (12)	
O3	1.1208 (4)	0.0946 (4)	0.1131 (2)	0.0611 (11)	
O4	1.2593 (4)	0.3245 (4)	0.0898 (2)	0.0569 (11)	

### Atomic displacement parameters (Å^2^)

**Table:**

	*U*^11^	*U*^22^	*U*^33^	*U*^12^	*U*^13^	*U*^23^
C1	0.041 (3)	0.043 (3)	0.057 (3)	0.004 (2)	0.010 (2)	0.003 (3)
C2	0.041 (3)	0.054 (4)	0.098 (5)	−0.001 (3)	0.019 (3)	0.001 (3)
C3	0.046 (3)	0.046 (3)	0.066 (4)	−0.001 (3)	0.022 (3)	0.003 (3)
C4	0.041 (3)	0.044 (3)	0.035 (3)	0.009 (2)	0.012 (2)	0.000 (2)
C5	0.036 (3)	0.063 (4)	0.044 (3)	−0.001 (3)	0.012 (2)	0.000 (3)
C6	0.037 (3)	0.044 (3)	0.049 (3)	0.005 (2)	0.022 (2)	0.004 (3)
C7	0.062 (4)	0.051 (3)	0.057 (3)	0.001 (3)	0.029 (3)	0.003 (3)
C8	0.089 (5)	0.056 (4)	0.073 (4)	−0.013 (4)	0.045 (4)	−0.018 (4)
C9	0.066 (4)	0.100 (6)	0.063 (4)	−0.003 (4)	0.035 (3)	−0.020 (4)
C10	0.067 (4)	0.082 (5)	0.053 (4)	0.019 (4)	0.034 (3)	0.014 (4)
C11	0.058 (4)	0.058 (4)	0.070 (4)	0.005 (3)	0.034 (3)	0.011 (3)
C12	0.040 (3)	0.046 (3)	0.047 (3)	0.005 (2)	0.016 (2)	0.001 (3)
C13	0.034 (3)	0.057 (3)	0.054 (3)	0.003 (3)	0.014 (2)	0.004 (3)
C18	0.059 (3)	0.057 (4)	0.046 (3)	0.007 (3)	0.005 (3)	−0.004 (3)
C14	0.039 (3)	0.062 (4)	0.058 (3)	−0.003 (3)	0.007 (3)	0.005 (3)
C15	0.063 (4)	0.066 (4)	0.053 (3)	0.002 (3)	0.014 (3)	−0.016 (3)
C16	0.068 (4)	0.066 (4)	0.068 (4)	−0.001 (3)	0.035 (3)	−0.015 (3)
C17	0.046 (3)	0.053 (3)	0.065 (4)	−0.013 (3)	0.024 (3)	−0.016 (3)
N1	0.038 (2)	0.038 (2)	0.048 (2)	0.0001 (19)	0.0159 (19)	0.006 (2)
N2	0.036 (2)	0.041 (2)	0.035 (2)	0.0063 (18)	0.0121 (17)	0.0054 (18)
O1	0.047 (2)	0.046 (2)	0.125 (4)	−0.0046 (19)	0.030 (2)	0.012 (2)
O2	0.078 (3)	0.040 (2)	0.099 (3)	0.008 (2)	0.040 (2)	0.024 (2)
O3	0.054 (2)	0.056 (2)	0.058 (2)	0.009 (2)	0.0075 (18)	0.009 (2)
O4	0.0379 (19)	0.080 (3)	0.050 (2)	−0.0048 (19)	0.0159 (16)	−0.015 (2)

### Geometric parameters (Å, °)

**Table:**

C1—N2	1.466 (6)	C9—C10	1.366 (9)
C1—C6	1.511 (7)	C9—H9	0.9300
C1—C2	1.542 (8)	C10—C11	1.386 (8)
C1—H1	0.9800	C10—H10	0.9300
C2—O1	1.433 (7)	C11—H11	0.9300
C2—H2A	0.9700	C12—C17	1.365 (7)
C2—H2B	0.9700	C12—O4	1.378 (6)
C3—O2	1.194 (6)	C12—C13	1.391 (7)
C3—O1	1.332 (6)	C13—C14	1.364 (8)
C3—N1	1.402 (7)	C13—H13	0.9300
C4—O3	1.195 (6)	C18—N2	1.469 (6)
C4—N1	1.410 (6)	C18—H18A	0.9600
C4—C5	1.484 (7)	C18—H18B	0.9600
C5—O4	1.417 (6)	C18—H18C	0.9600
C5—H5A	0.9700	C14—C15	1.355 (9)
C5—H5B	0.9700	C14—H14	0.9300
C6—C11	1.378 (7)	C15—C16	1.373 (8)
C6—C7	1.404 (8)	C15—H15	0.9300
C7—C8	1.372 (8)	C16—C17	1.369 (7)
C7—H7	0.9300	C16—H16	0.9300
C8—C9	1.374 (9)	C17—H17	0.9300
C8—H8	0.9300	N1—N2	1.410 (5)
			
N2—C1—C6	110.6 (4)	C9—C10—H10	119.7
N2—C1—C2	109.3 (4)	C11—C10—H10	119.7
C6—C1—C2	114.7 (5)	C6—C11—C10	121.1 (6)
N2—C1—H1	107.3	C6—C11—H11	119.5
C6—C1—H1	107.3	C10—C11—H11	119.5
C2—C1—H1	107.3	C17—C12—O4	125.0 (5)
O1—C2—C1	112.2 (4)	C17—C12—C13	119.5 (5)
O1—C2—H2A	109.2	O4—C12—C13	115.5 (5)
C1—C2—H2A	109.2	C14—C13—C12	119.4 (5)
O1—C2—H2B	109.2	C14—C13—H13	120.3
C1—C2—H2B	109.2	C12—C13—H13	120.3
H2A—C2—H2B	107.9	N2—C18—H18A	109.5
O2—C3—O1	119.9 (5)	N2—C18—H18B	109.5
O2—C3—N1	124.9 (5)	H18A—C18—H18B	109.5
O1—C3—N1	115.2 (5)	N2—C18—H18C	109.5
O3—C4—N1	122.3 (5)	H18A—C18—H18C	109.5
O3—C4—C5	124.0 (5)	H18B—C18—H18C	109.5
N1—C4—C5	113.6 (4)	C15—C14—C13	121.3 (5)
O4—C5—C4	110.6 (4)	C15—C14—H14	119.4
O4—C5—H5A	109.5	C13—C14—H14	119.4
C4—C5—H5A	109.5	C14—C15—C16	119.1 (5)
O4—C5—H5B	109.5	C14—C15—H15	120.5
C4—C5—H5B	109.5	C16—C15—H15	120.5
H5A—C5—H5B	108.1	C17—C16—C15	120.8 (6)
C11—C6—C7	117.3 (5)	C17—C16—H16	119.6
C11—C6—C1	124.1 (5)	C15—C16—H16	119.6
C7—C6—C1	118.6 (5)	C12—C17—C16	119.8 (5)
C8—C7—C6	121.4 (6)	C12—C17—H17	120.1
C8—C7—H7	119.3	C16—C17—H17	120.1
C6—C7—H7	119.3	C3—N1—N2	121.0 (4)
C7—C8—C9	120.2 (6)	C3—N1—C4	122.8 (4)
C7—C8—H8	119.9	N2—N1—C4	116.0 (4)
C9—C8—H8	119.9	N1—N2—C1	109.0 (4)
C10—C9—C8	119.5 (6)	N1—N2—C18	110.8 (4)
C10—C9—H9	120.3	C1—N2—C18	114.0 (4)
C8—C9—H9	120.3	C3—O1—C2	126.3 (4)
C9—C10—C11	120.6 (6)	C12—O4—C5	116.7 (4)
			
N2—C1—C2—O1	37.4 (7)	C15—C16—C17—C12	1.0 (10)
C6—C1—C2—O1	−87.4 (6)	O2—C3—N1—N2	165.9 (5)
O3—C4—C5—O4	2.4 (7)	O1—C3—N1—N2	−14.2 (7)
N1—C4—C5—O4	−179.3 (4)	O2—C3—N1—C4	−18.8 (9)
N2—C1—C6—C11	−82.9 (6)	O1—C3—N1—C4	161.1 (5)
C2—C1—C6—C11	41.2 (7)	O3—C4—N1—C3	0.8 (8)
N2—C1—C6—C7	94.9 (6)	C5—C4—N1—C3	−177.6 (5)
C2—C1—C6—C7	−141.0 (5)	O3—C4—N1—N2	176.3 (4)
C11—C6—C7—C8	−0.6 (8)	C5—C4—N1—N2	−2.0 (6)
C1—C6—C7—C8	−178.5 (5)	C3—N1—N2—C1	49.4 (6)
C6—C7—C8—C9	2.2 (9)	C4—N1—N2—C1	−126.2 (4)
C7—C8—C9—C10	−2.5 (10)	C3—N1—N2—C18	−76.8 (6)
C8—C9—C10—C11	1.2 (10)	C4—N1—N2—C18	107.6 (5)
C7—C6—C11—C10	−0.6 (8)	C6—C1—N2—N1	68.9 (5)
C1—C6—C11—C10	177.1 (5)	C2—C1—N2—N1	−58.2 (5)
C9—C10—C11—C6	0.3 (9)	C6—C1—N2—C18	−166.8 (4)
C17—C12—C13—C14	−2.7 (8)	C2—C1—N2—C18	66.1 (6)
O4—C12—C13—C14	178.3 (5)	O2—C3—O1—C2	169.7 (6)
C12—C13—C14—C15	1.2 (9)	N1—C3—O1—C2	−10.2 (9)
C13—C14—C15—C16	1.4 (9)	C1—C2—O1—C3	−3.0 (9)
C14—C15—C16—C17	−2.5 (10)	C17—C12—O4—C5	21.0 (8)
O4—C12—C17—C16	−179.5 (5)	C13—C12—O4—C5	−160.0 (5)
C13—C12—C17—C16	1.6 (9)	C4—C5—O4—C12	−90.5 (6)

### Hydrogen-bond geometry (Å, °)

**Table:**

*D*—H···*A*	*D*—H	H···*A*	*D*···*A*	*D*—H···*A*
C7—H7···O4^i^	0.93	2.53	3.394 (7)	154
C9—H9···O3^ii^	0.93	2.61	3.383 (8)	141
C13—H13···Cg2^iii^	0.93	2.86	3.715 (6)	153
C18—H18B···Cg3^i^	0.96	2.74	3.672 (6)	165

Symmetry codes: (i) −*x*+2, −*y*+1, −*z*; (ii) −*x*+2, *y*+1/2, −*z*+1/2; (iii) *x*+1, *y*, *z*.

## References

[bb1] Altomare, A., Burla, M. C., Camalli, M., Cascarano, G. L., Giacovazzo, C., Guagliardi, A., Moliterni, A. G. G., Polidori, G. & Spagna, R. (1999). *J. Appl. Cryst.* **32**, 115–119.

[bb2] ChemAxon (2008). *MarvinSketch* ChemAxon Kft, Budapest, Hungary. URL: http://www.chemaxon.com.

[bb3] Cremer, D. & Pople, J. A. (1975). *J. Am. Chem. Soc.* **97**, 1354–1358.

[bb4] Enraf–Nonius (1994). *CAD-4 EXPRESS* Enraf–Nonius, Delft, The Netherlands.

[bb5] Farrugia, L. J. (1997). *J. Appl. Cryst.* **30**, 565.

[bb6] Farrugia, L. J. (1999). *J. Appl. Cryst.* **32**, 837–838.

[bb7] Harms, K. & Wocadlo, S. (1995). *XCAD4* University of Marburg, Germany.

[bb8] Iulek, J. & Zukerman-Schpector, J. (1997). *Quim. Nova*, **20**, 433–434.

[bb9] Nardelli, M. (1995). *J. Appl. Cryst.* **28**, 659.

[bb10] Rodrigues, A., Olivato, P. R. & Rittner, R. (2005). *Synthesis*, pp. 2578–2582.

[bb11] Rodrigues, A., Olivato, P. R., Zukerman-Schpector, J. & Rittner, R. (2006). *Z. Kristallogr.* **221**, 226–230.

[bb12] Sheldrick, G. M. (2008). *Acta Cryst.* A**64**, 112–122. 10.1107/S0108767307043930 18156677

